# Asymptomatic *Plasmodium* infection and associated factors among pregnant women in the Merti district, Oromia, Ethiopia

**DOI:** 10.1371/journal.pone.0248074

**Published:** 2021-03-25

**Authors:** Bereket Wake Subussa, Teferi Eshetu, Teshome Degefa, Musa Mohammed Ali

**Affiliations:** 1 Department of Medical Laboratory Science, College of Health Science, Arsi University, Assella, Ethiopia; 2 School of Medical Laboratory Science, College of Health Sciences, Jimma University, Jimma, Ethiopia; 3 Schools of Medical Laboratory Science, College of Medicine and Health Sciences, Hawassa University, Hawassa, Ethiopia; Federal University of Agriculture, Abeokuta, NIGERIA

## Abstract

**Background:**

Asymptomatic *Plasmodium* infection (API) that occurs during pregnancy increases the risk of stillbirths, abortion, premature delivery, and low birth weight. API also hinders the control and prevention of malaria as infected hosts serve as silent reservoirs for transmission of *Plasmodium species* in the community.

**Objective:**

The aim of this study was to determine the prevalence of API and associated factors among pregnant women. This community-based cross-sectional study was conducted at Merti district, Oromia, Ethiopia among 364 pregnant women from March to September 2018.

**Methods:**

Sociodemographic and obstetrics features were collected using a structured questionnaire. About 2ml of blood was collected from participants to detect *Plasmodium* species, gametocyte carriage rate, parasite density, and anemia.

**Results:**

The prevalence of API among pregnant women was 3.6%. The proportion of *Plasmodium falciparum* and *Plasmodium vivax* was 6(46.2%) and 7(53.8%) respectively. Out of 13 *Plasmodium* species identified, Gametocyte carriage rate was 4(30.7%). The geometric mean density of the asexual stage of the parasites was 994.7(interquartile [IQR], 320 to 2200) parasites/ul. The geometric mean gametocyte density was 303.3 (interquartile range [IQR], 160 to 600). The proportion of anemia among *Plasmodium*-infected participants was 12(92.3%). Previous infection by *Plasmodium* species (AOR = 5.42; 95% CI: 1.19–29.03, *p* = 0.047), lack of insecticide-treated bed net use (AOR = 6.52; 95% CI: 1.17–36.44, *p* = 0.032), and living close to stagnant water (AOR = 4.18; 95% CI (1.12–17.36, *p* = 0.049) were significantly associated with API. Anemia was significantly higher among *Plasmodium*-infected than non-infected pregnant women (*x*^2^ = 27.62, *p* <0.001).

**Conclusion:**

In the current study, a relatively high prevalence of API was detected among pregnant women. Identifying API in the community is important to prevent the unwanted outcomes of *Plasmodium* infection and its transmission.

## Introduction

Malaria is an infectious disease caused by a protozoan parasite of the genus *Plasmodium*. *Plasmodium falciparum* is the most prevalent and causes most of the fatal infections in Africa [1]. Infection occurring outside of Africa is mostly due to *Plasmodium vivax*, as it is capable to develop in a wider range of temperatures [1]. According to the World Health Organization (WHO) report of 2018, *P*. *falciparum* is the most prevalent in the African, South-East Asia, Eastern Mediterranean, and the Western Pacific Regions. However, *P*. *vivax* is the predominant parasite in the WHO Region of the Americas, representing 75% of malaria cases [2]. There are also growing pieces of evidence that show the high transmission of *P*. *vivax* in Africa [3].

Approximately, 228 million malaria cases and 405000 deaths occurred worldwide in 2018; most of the malaria-related cases and deaths were from Africa. Between 2015 and 2018, among malaria-endemic countries, only 31 of them were on the way to reduce the incidence by about 40% or more by the year 2020 [2]. More than 10 million pregnant women in moderate and high *Plasmodium* transmission countries were exposed to *plasmodium* infection in 2018. Out of 11 million pregnant women exposed to *Plasmodium* infections in the year 2019 give birth to about 872000 babies with low birth-weight [2]. About 30−40 *Anopheles* species are responsible for the transmission of malaria. Among them, *Anopheles arabiensis* is the primary vector of malaria in Ethiopia [4, 5].

Malaria during pregnancy affects the mother, fetus, and neonates [6]. It increases the risk of stillbirths, spontaneous abortion, premature delivery, and low birth weight [7]. In the African continent, where the greatest burden of malaria occurs, about 30 million women living in malaria-endemic regions become expectant each year. Malaria is a threat to these women and their babies, with up to 200,000 newborn deaths occurring each year as a result of malaria in pregnancy [7]. Although patients with subclinical infections do not present with malaria symptoms, they contribute to the cycle of transmission in a population. The relative contribution of sub-clinical infection has considerable implications for the design and use of elimination diagnostics [8].

In malaria-endemic areas, a significant proportion of individuals have an asymptomatic infection with *Plasmodium* species among which pregnant women are at higher risk [9]. In asymptomatic parasitemia, the person carries *Plasmodium* parasites in their bloodstream, but due to partial immunity, the parasites are incapable of inducing symptoms to the affected individual. Nevertheless, the infected person could transfer the parasites to other individuals via mosquito bite [10].

In areas where the transmission of malaria is seasonal, during the dry season *Plasmodium* species survive in the bloodstream of the asymptomatic persons. This allows malaria to resume to the population during the wet season [11]. During the following pregnancies, however, anti-VAR2CSA (Variant 2 chondroitin sulfate A) antibodies can be found in pregnant women’s circulation, giving them protective immunity against these variant parasites [11].

Asymptomatic malaria can be observed in both stable endemic areas and unstable transmission areas [12, 13]. However, much attention has been given to symptomatic *Plasmodium* infections, relatively little attention has been paid to asymptomatic malaria. Although it is difficult to define asymptomatic malaria because of the lack of standard diagnostic criteria, the most widely used criteria include the presence of parasites in peripheral thick blood smears, an axillary temperature below 37.5°C, and the absence of malaria-related symptoms [14].

In Ethiopia, there are different data on the prevalence of asymptomatic malaria and risk factors at the institutional level, but there is scarce data on asymptomatic malaria among pregnant women at the community level in general and particularly in the current study area. National Malaria Control and Elimination Programs are geared towards the protection of pregnant women living in malaria-endemic zones because of their reduced immunity [15]. Therefore, the present study was designed to determine the prevalence of asymptomatic *Plasmodium* infection (API) and associated factors during pregnancy.

## Materials and methods

### Study design and area

A community-based cross-sectional study was conducted to determine the prevalence and predictors of API among pregnant women in Merti district, Oromia, Ethiopia from March to September 2018. Merti district is located in Southeastern Oromia; it lies in a tropical climatic zone with an altitude of 1,000–3,280 meters above sea level, an average annual temperature of 26°C and rainfall of 600mm. The District consists of 22 villages, of which 17 have been known as malarious villages with an intense transmission pattern. The total population of Merti district was116, 822, of whom 60,257 were men and 56,565 were women; 22,539 of its population were urban dwellers [16].

### Study population

All pregnant women living in the selected Village of Merti district during the study period and fulfill the inclusion criteria.

### Inclusion and exclusion criteria

Pregnant women without signs and symptoms of malaria (fever, chills, rigor, nausea, vomiting, headache, joint/muscle pains, and anorexia) within the last 48 hours, axillary temperature ≤ 37°c, permanent residents in the study area and those willing to participate in the study, and signed the informed consent were included. Pregnant women who had taken antimalarial drugs in the last two weeks before the study period were excluded from the study.

### Sample size determination

The sample size was determined using a single population proportion formula using the following assumptions: previous prevalence of asymptomatic malaria among pregnant women reported from Arba Minch, Ethiopia (9.4%) [17]; 95% confidence interval, 3% margin of error, 10% non-response. The calculated sample size was 400. Merti woreda has 22 kebeles, of which 11 kebeles were randomly selected for this study by using a simple random sampling method. Since the total number of asymptomatic pregnant women found in selected kebeles during the study period was less than the calculated sample size, all (N = 364) asymptomatic pregnant women were included in the study. The distribution of pregnant women based on where they belong (kebele) is as follows: Worsha kona (n = 11), Woticha dole (n = 18), Abomsa 01 (n = 37) Ashe (n = 13), Wataro (n = 45), Shemo (n = 12), Hella-kiyya (n = 22), Abomsa 02 (n = 28), Gologota (n = 96) Dembeka-iftu (n = 52), Dembeka-gadjele (n = 30).

### Variables of the study

#### Dependent variables

Prevalence of API among pregnant women.

#### Independent variables

Age, Residence, Gestational age, Previous infection with *Plasmodium*, Living close to stagnant water, Indoor residual spray, use of insecticide-treated bed net, Gravida, ANC attendance, Anemia.

### Data collection

A structured questionnaire was used to collect sociodemographic and obstetric data. Information collected includes age, number of children, educational status, knowledge of malaria prevention, ownership of ITN, sleeping under the ITN, and use of the IRS.

About 2ml of the blood sample was collected from study participants from a peripheral vein into an Ethamine Diamine Tetra acetic Acid (EDTA) tube for preparation of thick and thin blood film. Rapid diagnostic test (RDT) and microscopy were used for the detection of *Plasmodium* species. Thick and thin blood smears were made on the same slide, fixed by methanol, air-dried, and transported to Abomsa and Gologota Health centers. The slides were stained with 10% Giemsa for 15 minutes and screened for the presence of *Plasmodium* species by using a microscope. RDT was performed according to the manufacturer’s manual.

Asexual parasite density per microliter (μl) of blood was determined by counting the number of parasites per 200 white blood cells on a thick blood film assuming a total standard white blood cell (WBC) count of 8000/μl [18]. The degree of parasite density was graded as mild, moderate, and severe when the counts were between 1–999 parasites/μl, 1000–9999/μl, and >10,000/μl respectively, following the method described elsewhere [19].

Gametocyte density was quantified against 500 leukocytes in the thick blood films. This was converted to the number of gametocytes per microliter of blood, assuming a standard approximation of leukocyte count of 8,000/ μl. A smear was considered negative if no parasites were seen after viewing 100 high-powered fields.

To determine anemia, two packed cell volume was used. Briefly, two heparinized capillary tubes of a 4-5cm column were filled with blood, one end of the capillary tube was sealed with plasticin, and samples were assembled in the Centrifuge (hematocrit machine) and spun at 5000 revolutions per minute for 5 minutes. The Packed cell volume (PCV) was read using Hawksleys microhematocrit reader. A study participant was considered anemic if the value of PCV was below 33%, according to WHO recommendation [20, 21].

### Data processing and analysis

Data were coded, entered into Epidata version 3.1, cleaned, and analyzed using SPSS version 20.0. Both descriptive and inferential statistics were used to analyze the data. The frequency was used to determine the prevalence of API among pregnant women. A bivariate and multivariable regression model was used to assess factors associated with asymptomatic malaria. A chi-square test was used to determine the association between malaria and anemia among pregnant women. Prevalence figures were calculated for the total study participants and the association between variables was calculated. A *p*-value of less than 0.05 was considered statistically significant.

### Quality control

The training was provided for data collectors on the data collection procedure. Originally the questionnaire was prepared in English; translated into Afan Oromo and Amharic language and then translated back to English. Prior to testing on actual study participants, all version of the questionnaire (English, Afan Oromo, and Amharic) were checked for accuracy and consistency. The questionnaire was also pretested on 5% of the total sample size and it is provided as S1 File. As part of validating the questionnaire, it was reviewed by two different individuals, pilot test was conducted, collected data was analyzed, internal consistency was checked, and finally the questionnaire was revised.

All laboratory materials such as rapid test kits, slides, thermometers, EDTA tube, and sample transporting system were checked for the expiration date, correct collection procedures, and samples as well as inbuilt control appearances by experienced laboratory professionals. One slide was examined by two laboratory professionals. The manufacturer’s instruction was strictly followed for the RDTs. All data were double entered to reduce data entry errors.

### Ethics approval and consent to participate

This study was ethically approved by Institutional Review Board of Institute of Health, Jimma University. Permission was obtained from the Arsi Zone Health Bureau and Merti Woreda Health Bureau. Written informed consent was obtained from each study participants after the purpose of the study was explained to them. The study was conducted in accordance with the Declaration of Helsinki.

## Results

### Sociodemographic and obstetric characteristics

Out of 364 pregnant women who participated in the current study, 121 (33.2%) had no formal education, 354 (97.3%) were married, and 160 (44%) were in the second trimester. The age of the study participants ranged from 16 to 40 years with a mean age of 28 years (**Table 1**).

**Table 1:** Sociodemographic and obstetric characteristics of the study participants, Merti district, Southeastern Oromia, March to September 2018 (N = 364).

Variables		Frequency n (%)
Age group in years	18–24	132 (36.3)
	25–34	165 (45.3)
	≥35	67 (18.4)
Education level	No formal education	121 (33.2)
	Primary education	177 (48.6)
	Above primary education	66 (18.1)
Marital status	Married	354 (97.3)
	Single	6 (1.7)
	Divorced	4 (1.1)
Occupation	Farmer	152 (41.8)
	Government employee	9 (2.5)
	Housewives	203 (55.8)
Gestation	First trimester	63 (17.3)
	Second trimester	160 (44.0)
	Third trimester	141 (38.7
Gravidity	Primigravidae	117 (32.1)
	Secondigravidae	145 (39.8)
	Multigravidae	102 (28.1
ANC Attendance	Yes	266 (73.0)
	No	98 (27.0)
Place of residence	Urban	103 (28.3)
	Rural	261 (71.7)

ANC: Antenatal care.

### Prevalence of API

The prevalence of API among pregnant women in the current study was 13(3.6%) 95% CI: [2.1, 6.01]. The proportion of *P*. *falciparum* and *P*.*vivax* was 6(46.2%) and 7(53.8%) respectively. Identical results were obtained by both RDT and microscope. Of the microscopically confirmed cases, 4(30.8%) had a gametocyte stage. The proportion of the gametocyte stage of *P*. *falciparum* was 3(50%), whereas the proportion of *P*. *vivax* gametocyte was 1(14.3%). The geometric mean density of the asexual stage of the parasites was 994.7(interquartile [IQR], 320 to 2200) parasites/ul. The geometric mean gametocyte density was 303.3 (interquartile range [IQR], 160 to 600). The majority of the study participants had mild parasitemia 11(84.6%), while 2(15.4%) had moderate parasitemia.

### Factors associated with API

Pregnant women who did not use insecticide-treated bed net (ITN), who had a previous history of *Plasmodium* infection, and who lived close to stagnant water (less than 1 km from vector breeding sites) were significantly associated with API (**Table 2**).

**Table 2:** Predictors of asymptomatic *Plasmodium* infection among pregnant women in Merti district, March-September 2018 (N = 364).

Variables		*Plasmodium infection* n (%)		COR (95% CI)	*p*-value	AOR (95% CI)^a^	*p*-value
		Yes	No				
Age in year	18–24	5 (3.8)	127 (96.2)	2.59(0.29–22.77)	0.388	4.05(0.38–42.58)	0.243
	25–34	7 (4.2)	158 (95.8)	2.92(0.35–24.32)	0.224	5.44(0.57–51.81)	0.140
	>35	1 (1.5)	66 (98.5)	1		1	
Residence	Rural	8 (3.1)	253 (96.9)	1.50(0.48–4.68)	0.183	1.52(0.38–5.88)	0.562
	Urban	5 (4.8)	98 (95.2)	1		1	
Gestational Age of pregnancy	1st trimester	3 (4.8)	60(95.2)	3.47(0.56–21.33)	0.179	2.95(0.41–21.33)	0.284
	2nd trimester	8 (5))	152 (95)	3.65 (0.76–17.5)	0.105	3.69(0.67–20.22)	0.132
	3^rd^ trimester	2 (1.4)	139(98.6)	1		1	
Previous infection by *Plasmodium*	Yes	11 (15.5)	60 (84.5)	5.43(1.18–24.8)	0.029	5.42(1.19–29.03)	0.047
	No	2 (0.7)	291 (99.4)	1		1	
Living close to stagnant water	Yes	10 (8.3)	111 (91.7)	6.41(1.73–23.7)	0.005	4.18(1.12–17.36)	0.049
	No	3 (1.2)	240 (98.8)	1		1	
ITN	Yes	2 (0.67)	298 (99.3)	1		1	
	No	11 (17.2)	53 (82.8)	5.1(1.16–23.37)	0.036	6.52(1.17–36.44)	0.032
IRS	Yes	5 (2)	249 (98)	1		1	
	No	8 (7.2)	102 (92.7)	1.52(0.48–4.73)	0.170	1. 75(0.44–6.57)	0.429
Gravidity of pregnancy	Primigravidae	4 (3.4)	113 (96.6)	1.16(0.25–5.43)	0.482	1.61(0.25–10.26)	0.312
	Secondigravidae	6 (4.1)	139(95.9)	1.42(0.34–5.83)	0.250	3.15(0.53–18.44)	0.203
	Multigravidae	3 (2.9)	99 (97.1)	1		1	
ANC attendance	Yes	5 (1.8)	261 (98.1)	1		1	
	No	8 (8.2)	90 (91.8)	4.61(1.14–15.57)	0.231	4.06(0.98–16.74)	0.252

COR: Crude odds ratio, CI: Confidence interval, AOR: Adjusted odds ratio, IRS: Indoor residual spray, ANC: Antenatal care:, ITN: Insecticide treated bed net.

^a^ variable with p-value <0.25 was considered for multivariable logistic regression.

### The association between API and anemia

The overall proportion of anemia among pregnant women in this study was 102(28%). There was a statistically significant association between API and anemia (*x*^2^ = 27.62, *p* = <0.001). The proportion of anemia among *Plasmodium*-infected and non-infected pregnant women was 12(92.3%) and 90(25.6%), respectively (**Table 3**). The majority of pregnant women with mild parasitemia 10(90.9%) were anemic. All pregnant women with moderate parasitemia were anemic.

**Table 3:** Association between asymptomatic *Plasmodium* infection and anemia among study participants.

Malaria status	Anemia		Chi-square	*p* -value
	Anemic	Normal		
Positive	12 (92.3)	1 (7.7)	27.62	0
Negative	90 (25.6)	261 (74.4)		
Total	102 (28.0)	262 (72.0)		

## Discussion

In the current study, the overall prevalence of API among pregnant women was 3.6%. This finding is lower compared to the results of similar studies conducted in South Ethiopia (9.1%) [17], Republic of Congo (7%) [22], Nigeria (22–38.8%) [23, 24], and Congo (10.8%) [25]. A systematic review and meta-analysis conducted in Ethiopia also estimated the high prevalence of asymptomatic malaria (7.83) [26]. The low prevalence of API among pregnant women in this study could be due to increased malaria control interventions in Ethiopia [27] or difference in malaria epidemiology between the study areas. The present study was conducted during a minor malaria transmission season which could also contribute to the lower prevalence of API among pregnant women reported in this study. On the other hand, the prevalence of API documented in this study is similar to the findings of a study conducted in Bangladesh, which reported a 2.3% prevalence of malaria among pregnant women [28]. The fact that we did not use molecular techniques which can detect submicroscopic *Plasmodium* infection could have been underestimated the prevalence of API. A study conducted in Colombia reported a high prevalence of submicroscopic *Plasmodium* infection (79%) by using a quantitative polymerase chain reaction [29].

There is growing evidence that shows the high transmission of *P*. *vivax* in Africa [3]. In the present study, the prevalence of *P*. *vivax* was slightly higher (53.8%) than *P*. *falciparum* (46.2%) which is in contrast to the national *Plasmodium* species composition which shows a higher proportion of *P*. *falciparum* than *P*. *vivax* [30]. Recent studies conducted in different parts of Ethiopia have also indicated a similar shift in *Plasmodium* species composition from *P*. *falciparum* predominance to *P*. *vivax* predominance [17, 31, 32]. This could be explained by the fact that the prevention and control activities of malaria in Ethiopia mainly focus on *P*. *falciparum* [33]. Treatment and diagnostic policies aimed exclusively on *P*. *falciparum* are far less efficient in controlling endemic *P*. *vivax* [3]. Other possible reasons may be climate variability or *P*.*vivax* might have developed resistance to chloroquine [34].

Malaria transmission largely depends on the presence of viable gametocytes in peripheral blood circulation, which are picked up by *Anopheles* mosquitoes during a blood meal [35, 36]. Gametocyte carriers are reservoirs of infection that play a key role in sustaining malaria transmission in the community [37]. In this study, the gametocyte carriage rate among pregnant women was 30.8%. The transmission of *Plasmodium* species from humans to mosquitoes requires the presence of gametocytes in the human peripheral blood. Thus, the detection of asymptomatic cases with circulating gametocytes signals their importance in maintaining sustained transmission in the study area. Indeed, the presence of asymptomatic cases is a big challenge for the management of elimination programs in the malaria-endemic area.

In the present study, ITN use, previous history of *Plasmodium* infection, and living close to vector breeding sites were the main predictors of API in the study area. Pregnant women who did not use ITN were 6.5 times more likely to have API as compared to those who used ITN. This finding is comparable with reports from the Southern part of Ethiopia [17] and Nigeria [24]. Educating pregnant women on the role of ITNs in preventing malaria will impact positively in reducing the prevalence of malaria. In the present study, we observed some individuals who used ITNs for a different purpose, for instance for the temporary storage of maize rather than using at night for mosquito control.

Pregnant women who lived close to the vector-breeding sites were 4 times more likely to have API as compared to those who lived away from the vector-breeding site. This is in agreement with studies conducted in West Arsi Zone [32] and the Southwestern part of Ethiopia [38]. There was also a statistically significant association between API and previous infection by *Plasmodium*. Individuals who had a previous history of *Plasmodium* infections were 5 times more like to have API compared to those with no previous history of malaria. These results underscore the need to further evaluate ways to optimize the management of API in pregnant women living in malaria-endemic areas. Even though gravidity was not significantly associated with API (*p*>0.05) there was a high proportion of API among pregnant women with two gravidities. In endemic areas, overtime pregnant women develop antibodies to *Plasmodium* variant surface antigen. This could protect pregnant women in subsequent pregnancies from *Plasmodium* infection [39].

Asymptomatic *Plasmodium* infection is one of the major causes of anemia in malaria-endemic settings. Our study also suggested that *Plasmodium* infections in pregnant women contribute to maternal anemia. Other studies in Ethiopia [17] and elsewhere [40–43] have also reported a significant association between API and anemia among pregnant women. The proportion of anemia among pregnant women in the current study (28.0%) is low compared to the findings reported from Nigeria (38.85%) [23]; however, it is high compared to report from India (23%) [44]. It might be difficult to attribute the proportion of anemia solely to API as other causes such as helminthiasis [45], malnutrition, HIV infection [46], and sickle cell anemia could be responsible for it.

## Conclusion

Asymptomatic *Plasmodium* infection and anemia during pregnancy is common the study area. Factors such as previous history of malaria, living close to stagnant water, and lack of ITN use were the main predictors of API among pregnant women. Appropriate treatment and control of API in the study area are required.

## Supporting information

S1 File Questionnaire. (PDF)

S2 File Raw data in SPSS. (SAV)

## Abbreviations

API: Asymptomatic *Plasmodium* infection
ITN: Insecticide treated bed net
WHO: World Health Organization
RDT: Rapid diagnostic test
WBC: White blood cell
IRS: Indoor residual spray
ANC: Antenatal care
