# Supplementary material for: Asymptomatic Plasmodium infection and associated factors among pregnant women in the Merti district, Oromia, Ethiopia
Source: PLoS One. 2021 Mar 25;16(3):e0248074. doi: 10.1371/journal.pone.0248074 (PMC7993780; doi:10.1371/journal.pone.0248074)
Supplement: S1 File — (PDF) [file pone.0248074.s001.pdf]

S1 File

Questionnaire (English version)

Please tick [√] where appropriate and give the appropriate response to each item as presented.

**Section A**	**Socio-Demographic information**	
S. Number	code	___________
1	Age	_____________
2	Educational level	1. No formal Education<br>2. Primary<br>3. Secondary<br>4. College<br>5. Higher<br>6. Graduated
3	Marital status	1. Single<br>2. Married<br>3. Divorced<br>4. Separated<br>5. Widowed
4	Occupation	1. Daily labour<br>2. Government<br>3. NGO<br>4. Farmer<br>5. Others
5	Residency	1.Urban<br>2.Rural

**Section B**	**Obstetric characteristics**	
6	Gravidity	1. Primigravidae<br>2. Secondgravidae<br>3. Multigravidae
7	Current gestational age of pregnancy	1. First trimester<br>2. Second trimester<br>3. Third trimester

8	Number of still birth/Abortion	1. Once<br>2. Twice<br>3. Above two<br>4. Not aborted
9	Did you follow the ANC, before delivery?<br>1. Yes<br>2. No	

**Section C**	**Possession/ownership of ITN**	
10	Do you have a mosquito bed net?	1. Yes<br>2. No
11	If yes, how many mosquito bed nets do you have?	1. One<br>2. Two<br>3. More than three
12	Did you sleep under a mosquito net (ITN) last night?	1. Yes<br>2. No
13	Which periods do you use the net?	1. All year round<br>2. During rainy season<br>3. During dry season<br>4. Others (specify).....
14	What are the benefits of ITN use?	1. To prevent malaria<br>2. To sleep soundly<br>3. To provide warmth<br>4. To prevent insects bites<br>5. Other (specify)......
**Section D**	**Use of indoor residual spray**	
15	Have you ever heard about IRS?	1. Yes<br>2. No
16	Did spray indoor residual spray on your home?	1. Yes<br>2. No

17	If yes to the above question, how often do you spray?	1. All year round<br>2. During rainy season<br>3. During dry season<br>4.Others (specify)......
18	Is there stagnant water around your home?<br><br>If yes,<br>Distance of home from the stagnant water	1. Yes<br>2. No<br><br>1. <1km<br>2. 5km<br>3. > 10km
**Section E**	**About *plasmodium* parasite infections**	
19	Have you been infected with malaria in the last one year?	1. Yes<br>2. No
20	Where did you get diagnosed?	1.Hospital<br>2.Health center<br>3.Health post<br>4.Not tested all
21	Did you utilize correctly all prescribed dose of antimalarial drugs?	1.Yes<br>2.No

Questionnaire (Amharic version)

መ ል ሶትን በሚቀጥ ለዉ ምልክት የ ረጋግጡ(√)

ክፍል ሀ:የስነ-ማህበራዊ ሁኔታ		
ተራ ቁጥር	መለያቁጥር	------------
1	ዕድሜ	……..
2	የትምህርት ደረጃ	1. ማንበብና መጻፍ የማይችል<br>2. አንደኛደረጃ<br>3. ሁለተኛደረጃ<br>4.ኮሌጅ(ዲፕሎማ)<br>5. ዩኒቨርሲቲ
3	የጋብቻ ሁኔታ	1. ያላገባ/ች<br>2. ያገባ/ች<br>3.የፈታ/ች<br>4. የተለያዩ<br>5. የሞተችበት/ባት
4	የመኖሪያ ቦታ	1. ከተማ<br>2. ገጠር
5	ስራዎት ምንድ ነዉ?	1. አርሶ ዓደር<br>2. ተቀጣሪ<br>3.የግልስራ<br>4. ስራአጥ<br>5.ሌላ(ይገለፅ)
6	የቤተሰብ ብዛት	1.አንድ<br>2.ሁለት<br>3.ሶስትናከዚያበላይ

ክፍል ለ: ስለእርግዝና		
7	ስንተኛ እርግዝናሽ ነዉ?	1.አንድ<br>2.ሁለት<br>3.ሶስትናከዚያበላይ
8	ከአሁንበፊት ዉርጃ አጋጥሞሽ ያዉቃል?	1.አዎ<br>2.አይደለም
9	እርግዝናሽ ስንት ወሩ ነዉ?	1.አንደኛ ሶስት ወራት<br>2.ሁለተኛ ሶስት ወራት<br>3.ሶስተኛ ሶስት ወራት

ክፍል ሐ:የአጎበርናየጸረምባኬሚካልአጠቃቀም		
10	የአልጋ አጎበር አለዎት?	1.አዎ<br>2.አይደለም
11	አዎን ካሉ ምን ያህል ነዉ?	1.አንድ<br>2.ሁለት<br>3.ሶስትናከዚያበላይ
12	በስንት ሰኣት ወደመኝታ ይሄዳሉ?	
13	የአልጋ አጎበር በመኝታ ሰኣት ይጠቀማሉ?	1.አዎ<br>2.አይደለም
14	በየትኛዉ ወቅት ነዉ የምትጠቀሙት?	1.ሁልጊዜ<br>2.ክረምት<br>3.በጋ<br>4.ሌላ...
15	የአልጋ አጎበር መጠቀሞት ምን ጥቅም ያስገኞሎታል?	1.ምባንለመከላከል<br>2.ሙቀትለማግኛት<br>3.በራሪነብሳትንለመከላከል<br>4.ሌላ.......
16	ስለ ጸረ ምባ ኬሚካል ርጨት ሰምቶ ያዉቃሉ?	1.አዎ<br>2.አይደለም
17	ቤቶዎትን የጸረምባ ኬሚካል አስረጨቶ ያዉቃሉ?	1.አዎ<br>2.አይደለም
ክፍል መ: ስለ ምባ በሽታ		

18	ባለፉት አንድ ዓመት ዉስጥ በምባ ተይዞ ያዉቃሉ?	1.አዎ<br>2.አይደለም
19	የት ነዉ የተመረመሩት?	1.ሆስፒታል<br>2.ጤናጣብያ<br>3..ጤናኬላ<br>4.አልታከምኩም
20	የተዘዞሎትን መዳንት በአግባቡ ተጠቅሞዋል?	1.አዎ<br>2.አይደለም
21	ያቆረ /የተኛ ዉሃ በአከባቢ አለ?	1.አዎ<br>2.አይደለም

Questionnaire (Afan Oromo version)

Deebii keessan mallattoo kanaan agarsiisaa [√]

**kutaa A**	**Oddeeffannoo qaama hirmaataa**	
1	Waggaa meeqa?	-----------------
2	Sadarkaa baruumsaa keessan?	1. Kanhinbaratin<br>2. Sadarkaa 1ffaa<br>3. Sadarkaa 2ffaa<br>4. Kolleejjii<br>5. kaneebbiffamtee
3	Haalagaa'elaa yeroo ammaa?	1. Kanhinheerumin<br>2. Kanheerumte<br>3. Kanhiiktee<br>4. Kanirraadu'e
4	Hojjaan keesan?	1. Dafqaanbulaa<br>2.Hojjata mootummaa<br>3. Mit-mootummaa<br>4. Qonnaanbulaa
5	Iddoon jireenyaa keeeessa?	1. magaalaa<br>2. baadiyaa

**Kutaaa B**	**Amalootaa ulfaa**	
6	Ulfi keesan meeqaffaadha?	1. jalqaba<br>2. lamaffaa<br>3. sadaffaa
7	Yeroo meeqa ulfi sirraadeebi'ee?	1.tokko<br>2.lama<br>3.lama oli<br>4. hindeebinee
8	Ulfi keessan ji'ameeqa?	1. marsaa tokkoffaa<br>2. marsaa lammaffaa<br>3. marsaa sadaffaa

9	Hordoffii da’umsa dura gootaa?	1.ni hordofa<br>2. hinhordofu

**kutaa C**	**Waa’ee Fayyadammummaa Aggobaraa**	
10	Aggoobara qabduu?	1. hinqaba<br>2. hinqabuu
11	Yooqabaatte, meeqa qabdaa?	1. tokko<br>2. lama<br>3. sadioli
12	Yeroo raftuu agoobara keessa raftee?	1. eeyyu<br>2. hinrafnee
13	Yeroo kami agoobara fayyadamtaa?	1. yeroo hundaa<br>2. yeroo roobaa<br>3. yeroo bonaa
14	FaayidaanAgoobaraamaalii?	1.dhukkuba-busaa ittisuuf<br>2. sagalee dhoorkuuf<br>3. hoo’a aargachuuf<br>4.bookeen offirraa dhoowwuuf
**Kutaa D**	**Fayyadammummaa keemikaala bookee busaa**	
15	Waa’ee keemikaala bookee balleesuuf biifamuu beektuu?	1. nibeeka<br>2. hinbeekuu
16	Ittifayyadamtee beektaa?	1. hinbeekaa<br>2. hinbeekuu
17	Yoobeektan ta’ee yeroo meeqaaf?	1.yeroo hundaa<br>2. yeroo gannaa<br>3. yeroo bonaa
18	Bishaan kuufame naannoo keessan jiraa?<br>Yoojiraate hangam fagaata?	1.jiraa<br>2. hinjiruu<br>1. 1km<br>2. 5Km

		3. 10Km oli.
**Kutaa E**	**Waa’ee dhukuba busaa**	
19	Kana dura dhukuba busaatiin qabamtee beektaa?	1. eeyyu<br>2. hin beekuu
20	Eessatti yaalamtee?	1.Hospital<br>2. buufata fayyaa<br>3.keellaa fayyaa<br>4.hin yaalamnee
21	Qoricha siif kenname seeran fayyadamtee?	1.eeyyee<br>2. hinfayyadamnee
